# Comparison of endopyelotomy and laparoscopic pyeloplasty for poorly functioning kidneys with ureteropelvic junction obstruction

**DOI:** 10.4103/0970-1591.30255

**Published:** 2007

**Authors:** Pratipal Singh, Rakesh Kapoor, Amit Suri, Kamal Jeet Singh, Anil Mandhani, Deepak Dubey, Aneesh Srivastava, Anant Kumar

**Affiliations:** Department of Urology, Sanjay Gandhi Post Graduate Institute of Medical Sciences, Lucknow, India

**Keywords:** Endopyelotomy, laparoscopic pyeloplasty, poorly functioning kidney

## Abstract

Endopyelotomy and laparoscopic pyeloplasty are established procedures for ureteropelvic junction obstruction (UPJO) and historically a high failure rate has been observed in poorly functioning units with UPJ obstruction. The aim of this study is to compare the results of laparoscopic pyeloplasty with endopyelotomy in poorly functioning renal units, i.e., GFR under 25 ml/min.

**Materials and Methods:**

Retrospective analysis of all the patients who underwent either laparoscopic pyeloplasty or endopyelotomy for ureteropelvic junction obstruction in poorly functioning units between January 1998 and June 2005 was done. Follow-up renal scans, done at three, six, 12 months and yearly thereafter, were studied. Success was defined as symptomatic relief and/ or improvement in function (10% over baseline) in renal scan.

**Results:**

There were 23 patients in the endopyelotomy group and 15 patients in the laparoscopic pyeloplasty group with mean age of 25.3 years (9-53) and 26 years (10-44), respectively. Mean pelvic volume was 41.2 8cc ± 9.5 and 39.1cc ± 9.85 in the endopyelotomy group and laparoscopic pyeloplasty group, respectively. Mean preoperative GFR was 17.4 ± 5.7 ml/min and 21 ± 4.5 ml/min in the endopyelotomy group and laparoscopic pyeloplasty group, respectively and mean postoperative GFR was 21 ± 3.5 ml/min and 22 ± 3.9 ml/min, respectively. Eighteen and 11 patients were symptomatic in ethe ndopyelotomy group and laparoscopic pyeloplasty group, respectively while symptomatic improvement was seen in 14 and 11 patients, respectively. Mean follow-up was 12 months in the laparoscopy group and 28 months in the endopyelotomy group. Success rate was better for laparoscopic surgery group (15/15 = 100%) than for endopyelotomy (18/23 = 78.26%).

**Conclusions:**

Though the improvement in renal function is less in patients with UPJO with poorly functioning kidneys undergoing endopyelotomy or laparoscopic pyeloplasty, laparoscopic pyeloplasty gives better results in the form of symptomatic relief; however, renal function remains stable whichever the approach chosen.

For the last few decades open pyeloplasty has been the gold standard of surgical treatment for ureteropelvic junction (UPJ) obstruction with a success rate exceeding 90% in the long term.[1] Inherent problems associated with large surgical incision and the need for cosmetic advantage brought in minimally invasive techniques e.g., percutaneous and endoscopic incision of the ureteropelvic junction known as endopyelotomy. These procedures though associated with lower morbidity, have a success rate about 15–20% lower than that of open pyeloplasty.[2,3] Endopyelotomy is associated with poorer outcome in patients with severe hydronephrosis, poor renal function or a crossing vessel, secondary UPJ obstruction and outcomes also depend on the type of energy used for the incision.[4–6] To minimize the morbidity associated with open pyeloplasty and to prevail over the uncertainty of results of minimally invasive approach, Schuessler *et al* first reported dismembered laparoscopic pyeloplasty in 1993.[7] Since then its efficacy and feasibility have been duplicated in terms of the outcome of the procedure with associated advantage of minimal invasion.[8] Laparoscopic pyeloplasty is limited by a steep learning curve and the technical challenges posed by intracorporeal laparoscopic suturing.

However, irrespective of the surgical procedures chosen, a high failure rate has been observed in poorly functioning units with UPJ obstruction.[9,10] Some authors have recommended nephrectomy for poorly functioning kidneys (GFR< 15 ml /min)[11] whereas others advocate renal salvage procedures even in such poorly functioning kidneys.[12]

We present retrospective nonrandomized comparison of the results of laparoscopic pyeloplasty and endopyelotomy in poorly functioning renal units, i.e., GFR less than 25 ml/min.

## MATERIALS AND METHODS

It is a retrospective analysis of records of patients from the hospital database who underwent either laparoscopic pyeloplasty or endopyelotomy for ureteropelvic junction obstruction (UPJO) in poorly functioning units between January 1998 and June 2005. One patient with zero relative function on renal scan who wished to undergo laparoscopic pyeloplasty was excluded. A total of 38 patients were analyzed, 23 in the endopyelotomy group and 15 in the laparoscopic pyeloplasty group. Eighteen patients in the endopyelotomy group and 11 patients in the laparoscopic pyeloplasty group had flank pain at presentation and the rest were incidentally diagnosed on evaluation of hydronephrosis detected at ultrasonography of abdomen for unrelated reasons. None of these had undergone any previous urologic or other surgery. No patient had any associated comorbidity. The decision to proceed with laparoscopic pyeloplasty or endopyelotomy was taken jointly by the physician and patient after explaining the details of both procedures and their possible outcomes to patients. The diagnosis was based on ultrasonography (USG), intravenous urography (IVU) and diuretic renogram (^99m^Tc99 DTPA) or Whitaker test in patients who had equivocal renogram findings. The GFR was calculated for each renal unit using t-zero method of diuretic renogram. The USG, IVU and renal scans were dome in all patients. Retrograde pyelogram was done in cases with poorly visualized units on IVU to define anatomy. A single operator measured the renal pelvic volume using ultrasonography.

Percutaneous antegrade endopyelotomy was performed through a posterior superior or middle calyx and the ureteropelvic junction was cut laterally down to fat using cold knife in standard fashion. The incised area was intubated with a 14/7 Fr endopyelotomy stent (Microvasive, Natick, Mass) /6/24 double J stent (Devon, India) antegradely and stent was left for two to six weeks. Nephrostomy was removed as soon as urine was clear, followed by removal of urethral catheter.

Laparoscopic transperitoneal dismembered Anderson-Hynes pyeloplasty was done using a three-port standard technique over a 6/26 Fr *double J stent*. Ureter and renal pelvis were adequately spatulated and three-suture intracorporeal freehand laparoscopic suturing anastomosis was done using 4/0 vicryl sutures on round body needle. Urethral catheter was removed on second postoperative day and abdominal drain tube was removed once the drainage reduced to less than 25 ml in 24h. The double J stent was removed after six weeks. Both procedures were performed by the same group of surgeons.

Follow-up renal scans, done at three, six, 12 months and yearly thereafter, were studied and last follow-up GFR was taken as postoperative GFR. Success was defined as symptomatic relief (based on subjective feeling of the patient) or improvement in function (10% over baseline, cutoff value taken from the literature based on clinically meaningful improvements in renal function) in renal scan.

Student's t-test was used to analyze the significance of difference between the group mean of pre and postoperative renal function (GFR) and pelvic volume.

## RESULTS

The demographic profile, mean preoperative and postoperative pelvic volume and GFR for both the procedures are summarized in the Table 1. Mean follow-up was 12 months in the laparoscopy group and 28 months in the endopyelotomy group. All patients were under follow-up till last follow-up. Mean preoperative GFR was 17.4 ± 5.7ml/min in the endopyelotomy group and 21 ± 4.5 ml/min in the lap pyeloplasty group and difference between the two groups was not statistically significant (*P*≥0.005). In the endopyelotomy group postoperative GFR improved to 21 ± 3.5 ml/min, however, this increase in GFR was not statistically significant (*P* ≥ 0.005). Similarly pos operative GFR also improved in the lap pyeloplasty group to 22 ± 3.9 ml/min without any statistical significance (*P*≥0.005). When compared with each other this increase in GFR was not significant statistically (*P*≥0.005) between the two groups. Symptomatic relief was observed more in the laparoscopic pyeloplasty group (11/11) in comparison to the endopyelotomy group (14/18) and this difference was statistically significant (*P*≤0.005).

**Table 1:** Patients' characteristics in the two groups

	Endopyelotomy	Laparoscopic pyeloplasty	*P* value
No. of patients	23	15	
Age	25.3 years (9–53)	26 years (10–44)	>0.005
Mean pelvic volume	41.28 cc + 9.5	39.1 cc + 9.85	>0.005
Preoperative mean GFR (ml/min)	17.4 + 5.7	21 ml + 4.5	>0.005
Postoperative mean GFR (ml/min)	21 + 3.5	22 + 3.9	>0.005
Symptomatic at presentation	18	11	
Symptomatic relief	14	11	<0.005
Success rate	78.26% (18/23)	100%	<0.005
Mean follow-up (months)	28 (12–54)	12 (9–24)	

There were no complications observed in the endopyelotomy *group.* In the laparoscopy group significant bleeding requiring transfusion occurred in one and three patients had increased drain output that prolonged the hospital stay to seven days. The reasons for increased drainage were improperly placed stent in one and hematuria and clot colic in two patients.

Symptomatic success rates were better for the laparoscopic surgery group (15/15 = 100%) than for endopyelotomy (18/23 = 78.26%) and renal function remained stable in both the groups with out any significant improvement.

## DISCUSSION

Over the last two decades the treatment approach to UPJ obstruction has evolved from open pyeloplasty to various minimally invasive procedures like endopyelotomy, acucise catheter incision, balloon dilatation and laparoscopic pyeloplasty. Irrespective of the surgical procedures chosen, changes in GFR are not significant in poorly functioning units with UPJ obstruction.[9,10] Some authors have recommended nephrectomy for poorly functioning kidneys (GFR< 15 ml /min).[9] However, other authors advocate renal salvage and reconstructive procedure even in such poorly functioning kidneys.[12] Open pyeloplasty has a high success rate (90-100%), but it carries morbidity in the form of postoperative pain and relative long convalescence.[13]

Endopyelotomy has become a reasonable alternative to open surgery for the treatment of UPJ obstruction. It is less invasive, has fewer functional and esthetic sequalae than open pyeloplasty and does not compromise the outcome of open surgery if that becomes essential. It has been observed that patients with a large volume pelvis, poorly functioning ipsilateral kidney, with associated crossing lower pole vessel or long ureteral strictures are poor candidates for endopyelotomy with a success rate to the tune of 50-60%.[9,10,14,15] It has also been observed that better the GFR, better the outcome of endopyelotomy in poorly functioning units.[16] Endopyelotomy is often not advised in kidneys with a GFR less than 20 ml/min, but there is paucity of literature in this regard.[11] In a recently published study by the success rate of antegrade endopyelotomy in patients with poorly or moderately functioning kidneys (n=3) was 66% (2/3 improved) and 50% with retrograde endopyelotomy (n=12) in comparison to the overall success rate of 56 and 70% with antegrade endopyelotomy and retrograde endopyelotomy, respectively; however, poor to moderate renal function (assessed as relative function not GFR) did not statistically reduce the overall success rate of endopyelotomy.[17] In our study we did not investigate patients prior to endopyelotomy for identification of crossing lower pole vessel and our overall success rate was 78.2% in poorly functioning units which is slightly higher than that of the recent literature;[17] however, it is lower than the success rate reported for normally functioning kidneys[18,19] (approximately 92%), which again reinforces the concept that the success rate of endopyelotomy is poorer in patients with poorly functioning kidneys.

Laparoscopic dismembered pyeloplasty is principally the same as open pyeloplasty. The diseased segment is excised and reduction of pelvis is done in both the procedures, hence the degree of hydronephrosis, length of stenotic segment or presence of crossing do not have an effect on success. Although technically more difficult, it shares a similar success rate as its open counterpart. Potential contraindications to laparoscopic pyeloplasty are presence of small intrarenal pelvis and previously failed open pyeloplasty due to high risk of perinephric scarring and devascularization of proximal ureter.[4]

Only two single-center studies have done head to head comparison of percutaneous endopyelotomy and laparoscopic pyeloplasty to the best of our knowledge.[18,19] In both these studies the overall success rate (symptom-free) of endopyelotomy was 92% (mean follow-up 16 months and 53.8 months) and of laparoscopic pyeloplasty was 100% (mean follow-up 16 months and 52.5 months); however, in both these studies the function of ipsilateral moiety was not taken as a separate parameter and success on the basis of renal function of ipsilateral moiety was not evaluated. In another study which retrospectively compared endopyeloplasty with endopyelotomy and laparoscopic pyeloplasty (mean follow-up 12, 20 and 31.4 months, respectively), the resolution of pain and relief of obstruction, respectively, were seen in 100 and 100% patients with endopyeloplasty, 93 and 88% with endopyelotomy and 93 and 100% patients who had laparoscopic pyeloplasty.[20] In our study comparing two minimally invasive options for UPJO, success rates were significantly better in terms of symptomatic relief for laparoscopic pyeloplasty (15/15 = 100%) than for endopyelotomy (18/23 = 78.26%) although mean follow-up in laparoscopic pyeloplasty was only 12 months in comparison to 28 months in the endopyelotomy group. This difference in follow-up of the two arms is negated by the fact that all the improvement or failures were observed within 12 months in both the groups.

Our study suggests that even in poorly functioning kidneys, laparoscopic pyeloplasty gives significant relief of symptoms, especially pain. Though both the procedures did not result in significant improvement in GFR, proper reduction could have contributed to better results with laparoscopic pyeloplasty than endopyelotomy.

Certain limitations were present in our study and the most important of these being the retrospective nonrandomized nature of study. The surgeon and patient preferences dictated the choice of procedure. Apart from this, symptomatic improvement was subjective and may not be given too much credence. Finally, the number of patients in each group was small. Despite these limitations, the study had comparable population groups and follow-up was based on renogram along with subjective outcomes.

We feel that laparoscopic pyeloplasty is a better option than endopyelotomy for patients with UPJO with poorly functioning kidneys. Since improvement in renal function is not significant with either of the procedures it should be offered to symptomatic patients with poorly functioning units who don't accept ablative procedures like nephrectomy at the first go.

## CONCLUSIONS

Though the improvement in renal function is less in patients with UPJO with poorly functioning kidneys undergoing endopyelotomy or laparoscopic pyeloplasty, laparoscopic pyeloplasty gives better results in the form of symptomatic relief; however, renal function remains stable whichever the approach chosen. Therefore, laparoscopic pyeloplasty can be offered to patients with UPJO with poorly functioning kidneys who may not accept ablative procedures initially.
